# First report of *Candida sojae* sepsis in a preterm infant; A new fungal threat in neonatal care

**DOI:** 10.22034/cmm.2024.345248.1565

**Published:** 2024-02-01

**Authors:** Canan Seren, Asuman Birinci

**Affiliations:** 1 Department of Neonatology, Ondokuz Mayıs University Faculty of Medicine, Samsun, Türkiye; 2 Department of Clinical Microbiology, Ondokuz Mayıs University Faculty of Medicine, Samsun, Türkiye

**Keywords:** *Candida sojae*, Case Report, Preterm, Septicemia

## Abstract

**Background and Purpose:**

Very low birth weight neonates are at high risk of invasive infections, including invasive candidiasis, which is associated with significant morbidity and mortality. Although *Candida albicans*, *Candida parapsilosis*, and *Candida tropicalis* are well-known fungal pathogens in these neonates, *Candida sojae* has been recently reported as a new agent of invasive candidiasis.

**Case report:**

This study aimed to report a very low birth weight preterm neonate who had septicemia due to *C. sojae*. In this neonate, immature immune and gastrointestinal systems, prolonged parenteral nutrition due to feeding intolerance, central venous catheter, and broad-spectrum antibiotic use were risk factors leading to sepsis with *C. sojae*. The neonate recovered completely with timely antifungal treatment.

**Conclusion:**

This case emphasized the need to be aware of *C. sojae* as an emerging neonatal pathogen in preterm infants.

## Introduction

Very low birth weight (VLBW) preterm neonates are at high risk of invasive infections due to their immature immune and gastrointestinal systems and multiple device use in the Neonatal Intensive Care Units (NICU). According to recent Vermont-Oxford Network data, the incidence of late-onset sepsis in VLBW neonates was 88.5 per 1,000 and 5% of these episodes were due to fungi [ 1 ]. *Candida* spp. can be transmitted to the neonate either from the colonized mother during vaginal delivery or the neonate might get colonized from the NICU environment [ 2 , 3 ]. This colonization might eventually result in invasive candidial infections, which is a significant risk factor for morbidity and mortality in this vulnerable group.

*Candida albicans*, *Candida parapsilosis*, and *Candida tropicalis* are well-known agents for invasive candidiasis (IC), but newer non-*albicans* species like *Candida glabrata*, *Candida krusei*, *Candida utilis*, *Candida pelliculosa*, and *Candida auris* are recognized as invasive fungal pathogens leading to IC in NICU [ 4 - 6 ]. Two recent reports have identified *Candida sojae* as an agent for IC [ 7 , 8 ]. The present study aimed to report a VLBW preterm neonate who had septicemia due to *C. sojae* and announce it as an emerging neonatal pathogen.

## Case Report

The neonate was born at 32 weeks of gestational age via Cesarean section, weighing 1,045 g from a G:1 P:1 mother following a pregnancy complicated by intrauterine growth restriction. His first- and fifth-min Apgar scores were 6 and 6, respectively. He was put on noninvasive ventilation in the NICU and an umbilical venous catheter (UVC) was inserted. 

On the third day of life, his clinical condition deteriorated. Sepsis workup showed an increase in serum C-reactive protein (CRP) levels and *Acinetobacter baumanii* grew in the blood culture (BD BACTEC^TM^ Blood Culture Media). The UVC was removed and he was given broad-spectrum antibiotics: Meropenem (20 mg/kg/dose, per 12 h), amikacin (12 mg/kg, per 36 h), and prophylactic fluconazole (3 mg/dose, twice weekly) intravenously for 14 days. During that period, he had feeding intolerance and was dependent on parenteral nutrition (PN) supplied from a peripheral vein. 

On the 33^rd^ day of life, the neonate had severe apnea leading to intubation. Sepsis work-up showed elevated serum CRP levels; therefore, empirical antibiotic therapy was started again with meropenem and amikacin. Clinical microbiology laboratory reported candidial growth in blood culture (BD BACTEC™ Blood Culture Media). Therefore, empirical antifungal therapy was started with amphotericin B deoxycholate (6 mg/kg/day, infused for 2 h). 

Two days after the sepsis signs, a subclavian venous catheter (SVC) was inserted under general anesthesia. Four days after the insertion of the SVC, laboratory results revealed candidial growth from the peripheral blood cultures again. Amphotericin B infusion time was prolonged up to 3 h, but a catheter lock could not be performed since the neonate was dependent on PN. The neonate had a fever and simultaneous blood cultures were obtained from the SVC and peripheral veins to diagnose central line-associated bloodstream infection. 

The blood culture obtained from SVC was positive. Gram staining and inoculation on blood and Eosin-methylene blue agars were performed. Yeast cells were observed in the Gram-stained preparation and yeast-like colonies were seen in the blood agar.

**Figure 1:**
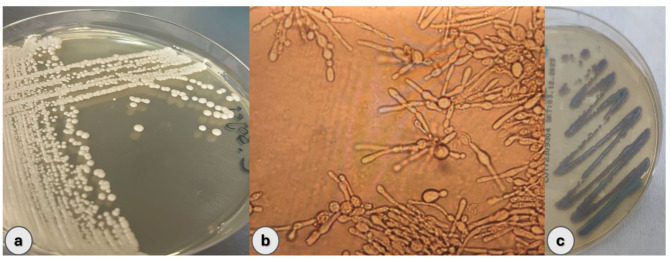
*Candida sojae* colonies on (a) Sabouraud dextrose agar, (b) Microscopic morphology of fungal structures on rice extract agar, and (c) Blue-purple appearance on chromogenic agar.

The mycology laboratory performed fungal cultivation on sabouraud dextrose agar (SDA), followed by chromogenic and rice extract agars. After incubation at 37 °C, yeast colonies were observed in SDA (Figure 1a) and the microscopy revealed Gram-positive yeast colonies (Figure 1b). 

On chromogenic agar (Becton Dickinson), the yeast growth was colorless at first, but after 72 h, a blue-purple color was observed, suggesting that the pathogen was *C. tropicalis* (Figure 1c). Since the microscopic appearance of the yeast on rice extract agar was different from *C. tropicalis*, fungal identification of *Candida* strain by mass spectrometry was performed by matrix-assisted laser desorption /ionization time-of-flight (MALDI-TOF) using Bruker MALDI-TOF Biotyper sirius (version 4.1, Bruker Daltonics, Bremen, Germany) device and the growing yeast was identified as *Candida sojae*. 

Following identification, antifungal susceptibility testing (MICRONAUT-AM Antifungal Agents Minimum inhibitory concentration [MIC], Bruker Daltonics, Germany) was performed. The MIC values of anidulafungin, micafungin, caspofungin, 5-Flucytosine, posaconazole, voriconazole, itraconazole, fluconazole, and amphotericin B were 0.002, 0.002, 0.06, <0.06, <0.008, <0.008, <0.03, 0.25, and 0.5 µg/ml, respectively.

The SVC was immediately removed. The neonate had leucopenia and thrombocytopenia during the course of *C. sojae* sepsis, necessitating platelet transfusions. Abdominal ultrasonography, echocardiography, and ophthalmological examinations did not show fungal dissemination. Moreover, the lumbar puncture did not show meningitis. Cranial ultrasound was normal. Fluconazole (12 mg/kg) was added to amphotericin B treatment due to lower MIC levels. 

**Figure 2:**
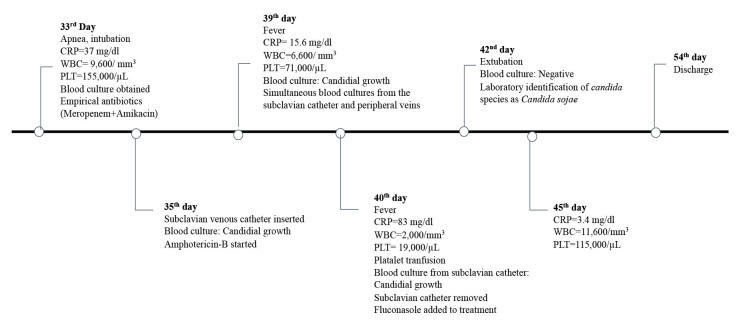
Timeline of the case. CRP: C-reactive protein, WBC: white blood count, PLT: platelet count

Since no guidelines exist for the treatment of *C. sojae*, antifungal therapy was guided by the clinical results, as well as serial CRP and complete blood count values. The clinical course was favorable, especially after the removal of the SVC. A timeline of the clinical course of the neonate is shown in Figure 2.

Amphotericin B and fluconazole were continued for 14 days after the first sterile blood culture. The neonate was discharged on the 54^th^ day of life. Cranial magnetic resonance imaging performed before discharge was normal. He is one year old now and did not have any infections after discharge. Fortunately, he made his catch-up growth and his neurodevelopment is compatible with his peers.

## Discussion

Newborns, especially VLBW preterm are at the highest risk of nosocomial infections, including sepsis, due to their immature immune system, underdeveloped intestinal barrier and microbiata, broad-spectrum antibiotic use, and invasive procedures in the NICU. Not surprisingly, this risk is inversely correlated with the gestational age and birth weight of the neonate [ 2 , 3 ]. 

Although *Candida* species, especially *C. albicans* are well-known pathogens for neonatal sepsis, recent publications have mentioned newer types of non-*albicans Candida*, like *C. utilis*, *C. pelliculosa*, and *C. auris* infections in VLBW infants [ 4 - 6 ]. *Candida sojae* is a new yeast species, first isolated from defatted soybean flakes in 1994 [ 7 ]. There are only two reports on *C. sojae* sepsis in the literature (one infant with short bowel syndrome and one adult), both occurring in patients with central venous catheters (CVCs) [ 8 , 9 ]. Our case is the first newborn in the literature with IC due to *C. sojae*.

This study could not identify the source of *C. sojae* in the patient. There was no case of candidial infection in the NICU when the patient was diagnosed. The main risk factors for IC infection in preterm are the presence of CVCs, assisted ventilation and intubation, prolonged antibiotic use (especially cephalosporins and carbapenems), PN, H_2_ receptor blocker use, and fungal colonization of the gastrointestinal tract [ 2 , 3 , 10 ]. This patient had an SVC, prolonged antibiotic, and PN use. Besides, he could get very little of the milk of his mother due to intestinal problems originating from intrauterine growth restriction and prematurity. Breast milk with its unique properties is protective against all neonatal infections. 

*Candida* pathogenesis advances through exposure, adherence, colonization, dissemination, and organ involvement; primarily the central nervous system, kidneys, heart, and eyes [ 3 ]. Central venous catheters are important risk factors for candidial sepsis and associated mortality as *Candida* species colonize them. 

Karadag- Öncel et al. reported that in infants below three months, failure of CVC removal was associated with a 20.5-fold increase in mortality in candidial sepsis. They concluded that removal of CVC significantly reduces mortality and is essential in the management of candidaemia [ 10 ]. *Candida* species can colonize CVCs and form biofilms [ 2 ]. Two patients in the literature with *C. sojae* sepsis also had CVCs [ 8 , 9 ]. 

The patient in the present study had a CVC inserted while he had fungemia and *C. sojae* was isolated from the SVC four days after the insertion, showing the colonization of CVC with the fungi. The SVC was immediately removed when the neonate had a fever for more than 24 hours and a positive blood culture sign was obtained from the SVC. This also implies that clinicians should think twice before inserting CVCs into patients with septicemia, since they might be colonized with these microorganisms, necessitating removal. 

The clinical presentation (fever, apnea, and not doing well) and acute phase markers of neonatal sepsis are not specific to the pathogen; therefore, clinicians have to work in coordination with the clinical microbiology department. Newer microorganisms should be kept in mind and detailed identification procedures should be performed when available. Usage of both conventional methods, namely SDA and microscopy, and more sophisticated methods, such as MALDI- TOF, led to the correct diagnosis in the present study, which implies that these methods should be used in harmony. 

Abdel-Haq reported that *C. sojae* was initially misidentified as *C. tropicalis* in an infant with IC, which we also observed in the present study [ 9 ]. Maybe *C. sojae* as a neonatal pathogen is not as rare as it is assumed, but remains unrecognized.

## Conclusion

Candida species are important etiological agents for nosocomial sepsis in VLBW preterm. Immature immune and gastrointestinal systems, lack of adequate breastfeeding, and prolonged antibiotic and PN use facilitated sepsis with *C. sojae* in this patient. As smaller preterm survive, NICUs will be faced with new opportunistic microorganisms causing invasive infections. Collaboration with clinical microbiology laboratories is essential to identify these new threats.
